# *N*-Acetyl Glucosamine Obtained from Chitin by Chitin Degrading Factors in *Chitinbacter tainanesis*

**DOI:** 10.3390/ijms12021187

**Published:** 2011-02-17

**Authors:** Jeen-Kuan Chen, Chia-Rui Shen, Chao-Hsien Yeh, Bing-Shiun Fang, Tung-Li Huang, Chao-Lin Liu

**Affiliations:** 1 Environment and Biotechnology Department, Refining and Manufacturing Research Institute, CPC Corporation, Taiwan, Chia-yi, Taiwan; E-Mail: 078450@cpc.com.tw (J.-K.C.); 075345@cpc.com.tw (B.-S.F.); 076538@cpc.com.tw (T.-L.H.); 2 Department of Medical Biotechnology and Laboratory Science, Chang Gung University, Kweishan, Taoyuan, Taiwan; E-Mail: crshen@mail.cgu.edu.tw; 3 Department of Chemical Engineering, Mingchi University of Technology, Taishan, New Taipei, Taiwan; E-Mail: chyen@mail.mcut.edu.tw; 4 Graduate School of Biochemical Engineering, Mingchi University of Technology, Taishan, New Taipei, Taiwan

**Keywords:** CDFs, *Chitinibacter tainanensis*, chitin, NAG, fermentation

## Abstract

A novel chitin-degrading aerobe, *Chitinibacter tainanensis*, was isolated from a soil sample from southern Taiwan, and was proved to produce *N*-acetyl glucosamine (NAG). Chitin degrading factors (CDFs) were proposed to be the critical factors to degrade chitin in this work. When *C. tainanensis* was incubated with chitin, CDFs were induced and chitin was converted to NAG. CDFs were found to be located on the surface of *C. tainanensis. N*-Acetylglucosaminidase (NAGase) and endochitinase activities were found in the debris, and the activity of NAGase was much higher than that of endochitinase. The optimum pH of the enzymatic activity was about 7.0, while that of NAG production by the debris was 5.3. These results suggested that some factors in the debris, in addition to NAGase and endochitinase, were crucial for chitin degradation.

## 1. Introduction

Chitin is the most abundant polysaccharide on the earth except cellulose, and it is a major component of most fungal cell walls, insect exoskeletons and the shells of crustaceans [1]. The chemical structure of chitin is a polymer of *N*-acetyl-d-glucosamine (NAG) linked by beta-glycosidic bond. Derivatives of chitin, including polysaccharides, oligosaccharides and monosaccharides, have performed several therapeutic activities such as immunomodulation [2,3], antitumor [4] osteoarthritis treatment [5], and so on [6–9]. Also, in the recent decade, NAG, the end hydrolytic product of chitin, has become an attractive biomaterial as food supplements and cosmetics [10–13].

Production of NAG by acid hydrolysis of chitin is expensive owing to low yield. The product manufactured by *N*-acetylation of glucosamine is not approval as a natural material due to the chemical process [14]. Several natural methods have been used to produce NAG. Using these methods, NAG is obtained by chitin hydrolysis using chitin degrading enzymes, containing endochitinases and *N*-acetylglucosaminidase (NAGase). The enzymes were usually isolated from microorganisms having strong chitinolytic activities [15,16]. Sashiwa *et al.* performed selective and efficient methods to produce NAG using a mixed enzyme preparation with high NAGase/endochitinase ratio [14,17,18].

In our laboratory, a novel chitin-degrading aerobe, *Chitinibacter tainanensis*, was isolated from a soil sample of southern Taiwan [19], and it was proven to produce NAG by degrading chitin. The yield was 0.75 g/g with α-chitin as substrate and was 0.98 g/g with β-chitin as substrate. A needle type crystal was produced after concentration and crystallization, and the purity was measured to be higher than 99%. The product was natural and was proven to be feasible for edible or cosmetic usage [9,12,20].

In this study, the mechanism by which *C. tainanensis* transforms chitin into NAG was elucidated by fermentation. A novel prospect for NAG obtained from chitin digestion by chitin degradation factors (CDFs) was also proposed.

## 2. Results and Discussion

### 2.1. Degradation of Chitin by “Chitin Degrading Factors”

**Figure 1.**
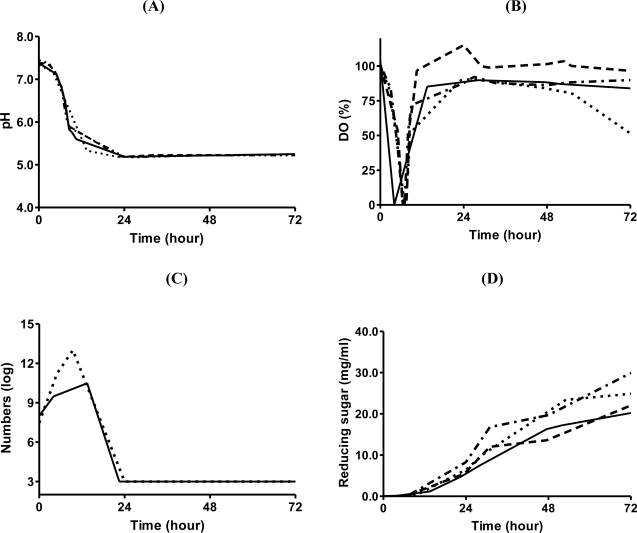
Parameters in fermentation. Each factor was evaluated more than once (indicated in brackets). (**A**) pH (triplicate); (**B**) DO (quadruplicate); (**C**) total aerobe number (duplicate); (**D**) reducing sugar (quadruplicate).

In order to understand the mechanism of chitin degradation by *C. tainanensis*, chitin degradation studies were performed in a 5 L fermenter. *C. tainanensis* was incubated in B-H broth containing 4% α-chitin powder. Dissolved oxygen (DO), pH, total aerobe count and reducing sugar were measured continuously for a period of 72 hours. The pH value of the broth decreased gradually from 7.4 to 5.3 in 14 hours (Figure 1A). DO decreased for 4 hours, after that, it increased up to 100% (Figure 1B). This implied that *C. tainanensis* proliferated rapidly for 14 hours, and some acidic metabolites were released and accumulated in the broth. After incubation, *C. tainanensis* was dead owing to the acidic broth, confirmed by counting of total aerobe number (Figure 1C).

It was interesting to notice that the concentration of reducing sugar in the broth was low within the first 14 hours; however, when *C. tainanensis* was incubated for longer than 14 hours, the level of reducing sugar increased over the incubation time of the tested period of 72 hours (Figure 1D). This suggested that some factors that were able to degrade chitin completely were produced simultaneously with *C. tainanensis* proliferation. The hydrolytic product was used as a carbon source for bacterial proliferation, and acidic metabolites were produced simultaneously. *C. tainanensis* was dead at pH 5.3; however, the assumed factors still existed in the broth. NAG, the final hydrolytic product of chitin degradation, was not consumed, but accumulated in the broth instead. The assumed factors were nominated “CDFs”.

### 2.2. Chitin Degrading Activity of Bacterial Debris

**Figure 2.**
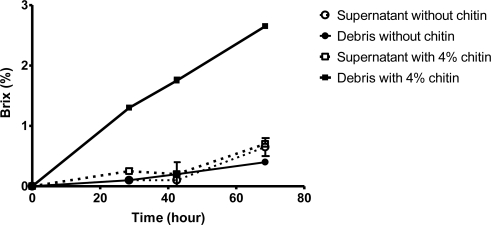
Chitin degrading effects of supernatant and bacterial debris isolated from the broth with or without 4% beta-chitin.

The bacterial debris was obtained by centrifugation, and it was suspended in 0.1 M NaOAc, pH 5.0. After 4% β-chitin was added, the brix increased dramatically in the reaction containing bacterial debris, while that in the reaction containing supernatant increased moderately. In another experiments, β-chitin was hardly degraded by the bacterial debris obtained from the incubation broth using glucose as a carbon source (Figure 2). β-chitin was more susceptible than α-chitin, and higher reducing sugar was produced [21]. NAG concentration was directly measured using a hand refractometer due to its high concentration in the broth. These results reflected that CDFs were located on the bacterial debris, and were induced by chitin powder.

In order to understand how CDFs hydrolyzed chitin, endochitinase and NAGase activities were measured. Both enzyme activities were detected in the bacterial debris. The specific activity of NAGase was much higher than that of endochitinase (data not shown). These results were in agreement with previous reports [14,17,18].

### 2.3. Chitin Degrading Effects of Fractions Isolated from the Bacterial Debris

**Figure 3.**
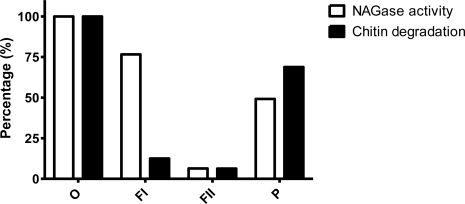
NAGase activity (□) and chitin degrading activity (▪) of different fractions isolated from the bacterial debris of *C. tainanensis.* Bacterial debris (O); dramatically shaken fraction (FI); surfactant extracted fraction (FII); residue (P).

Since assumed CDFs were located on the debris of *C. tainanensis*, it was reasonable to identify the structural information of CDFs. Bacterial debris (O), dramatically shaken fraction (FI), surfactant extracted fraction (FII) and residue (P) were obtained as described in the Experimental section. Chitin degrading effects and NAGase activities of the four fractions were compared, and those of bacterial debris were set as 100% (Figure 3). Chitin was not efficiently degraded in fraction FI which possessed high NAGase activity. Soluble surface components were almost extracted by shaking treatment due to low chitin degrading effect in fraction FII. In the residual fraction (P), chitin was efficiently degraded although lower NAGase activity existed in this fraction. These results suggested that CDFs strongly interacted with the bacterial surface, and were similar to the cellulosome concept in cellulose degradation [22]. In addition, NAGase activity did not play major roles in chitin degradation of *C. tainanensis.*

**Figure 4.**
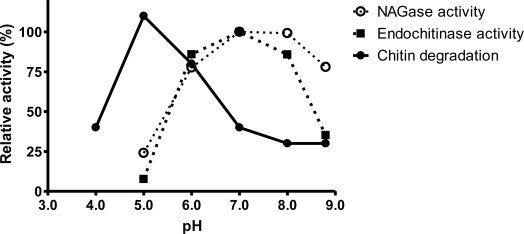
Relative activities of chitin degrading effect, NAGase and endochitinase in buffers of various pH.

Chitin degrading effects and enzymatic activities of the bacterial debris were also performed in buffers with different pHs. It was surprising that the optimum pH value of the chitin degrading effect was about 5.3, while those of endochitinase and NAGase isolated from the bacterial debris were about 7.0 (Figure 4). These results suggested that the mechanism of chitin degradation of *C. tainanensis* was novel and different from that of the enzymatic method published before. Some unknown factors not yet explored played important roles in chitin degradation.

## 3. Experimental Section

### 3.1. Materials

Powdered α-chitin and β-chitin used in this study were supplied from Charming and Beauty company (Taipei, Taiwan). Media for microorganism incubation were purchased from Difco Laboratories (Dentroit, MI). Colloidal chitin was prepared from α-chitin using the method of Jeniaux [23,24], and other chemicals used in this study were purchased from Sigma Chemical Co. (St. Louis, MO, USA).

### 3.2. Fermentation of *C. tainanensis*

The agar containing B-H broth, Difco Bushnell-Haas broth, and 0.2% colloidal chitin was used as a selective medium. The colonies with surrounding clear zones were obtained and grown in 400 mL Luria-Bertani medium at 30 °C overnight. A fresh overnight culture was transferred to a 5 L fermenter containing 4 L B-H broth supplemented with 4% α-chitin powder as a sole carbon source. The agitation rate and air feeding were set at 200 rpm and 4 L/min. All fermentations were performed at 30 °C. pH and DO were detected by an appropriate electrode, and the reducing sugar was analyzed with a spectrophotometer using potassium ferricyanide as substrate [25]. Numbers of bacteria in the fermenter were counted after spraying the diluted broth on nutrient agar following 48 hours incubation.

### 3.3. Preparation of Bacterial Debris and Its Components

*C. tainanensis* was incubated in B-H broth containing 4% β-chitin powder for 48 hours. Bacterial debris was collected by centrifugation, and was suspended in an equal volume of 0.1 M NaOAc, pH 5.0. Bacterial debris (O) was shaken dramatically to obtain the surface component in supernatant (FI) after centrifugation, and the pellet was further treated with 0.1% *N*-lauroylsarcosine to isolate the other component on the surface of bacterial debris (FII). After centrifugation, the residues were suspended in equal volume of 0.1 M NaOAc, pH 5.0 for test.

### 3.4. Analysis of Chitin Degrading Effect and Enzymatic Activity

Chitin was used as substrate to analyze the chitin degrading effect. Briefly, in the sterilized test tube, chitin was added to the bacterial debris or its components described above at a final concentration of 4%, and 10 μg/mL of tetracycline was added subsequently to prevent microorganism contamination. NAG released in the reaction was detected using a hand refractometer after centrifugation, and the result was expressed as brix (%). The yield was calculated from the equation: Yield (%) = NAG (g)/Chitin (g) added [14].

Endochitinase and NAGase activities were analyzed using 0.18 mM PNP-(GlcNAc)_3_ and 0.18 mM PNP-GlcNAc as substrate, respectively [7,20,24,26,27]. After 30 min incubation, NaOH was added to a final concentration of 0.1 M to terminate the reaction. The absorbance at 405 nm was measured using a spectrophotometer. When enzyme activity of insoluble component was analyzed, the insoluble component was removed by centrifugation before colorimetric measurement.

## 4. Conclusion

CDFs, chitin degrading factors, were proposed to describe the chitin degrading effect of *C. tainanensis*. When *C. tainanensis* was incubated with chitin, CDFs were induced and chitin was converted to NAG. After consumption of NAG, the medium became sour simultaneously, and the bacterium was dead, then NAG was accumulated in the medium. CDFs were found to be located on the surface of *C. tainanensis*. Chitin degradation and enzymatic activity measurement showed that chitin degradation of *C. tainanensis* was different from that of enzymatic method, and some unknown factors not yet explored played important roles in chitin degradation.
